# Optical coherence tomography

**DOI:** 10.1111/j.1365-2818.2012.03619.x

**Published:** 2012-09

**Authors:** A Gh Podoleanu

**Affiliations:** School of Physical Sciences, University of KentCanterbury, U.K.

**Keywords:** Broadband source, optical coherence tomography, partial coherence light, swept source, tunable laser, white light interferometry

## Abstract

The review provides a concise explanation of principles of operation of different optical coherence tomography methods. A comparative analysis of their advantages and disadvantages is presented in relation to specific applications. The review will assist the reader in making an educated choice on the most suitable optical coherence tomography method to be used in a particular application.

## Introduction

**Fig. 1:**
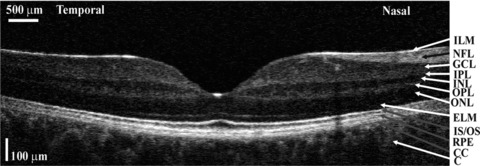
Cross-section image of the retina using an *en face* time domain OCT system driven by a large bandwidth source (Cucu *et al*., 2006). Lateral size: 7.5 mm, vertical size is along depth, 0.725 mm measured in the retina. ILM, inner limiting membrane; NFL, nerve fibre layer; GCL, ganglion cell layer; IPL, inner plexiform layer; INL, inner nuclear layer; OPL, outer plexiform layer; ONL, outer nuclear layer; ELM, external limiting membrane; IS/OS, junction between the inner and outer photoreceptors; RPE, retinal pigment epithelium; CC, choriocapillaris; C, choroid.

Optical coherence tomography (OCT) is a noninvasive high resolution optical imaging technology based on interference between signal from an object under investigation and a local reference signal. OCT can produce in real time a cross-section image of the object, i.e. a two-dimensional image in the space (lateral coordinate, axial coordinate). When applied to image the retina of the human eye, confocal microscopy is limited by the compound effect of the low numerical aperture of the eye and its aberration. In confocal microscopy, both lateral and axial resolutions are determined by the numerical aperture of the microscope objective. In OCT, the axial resolution is mainly determined by the optical source and therefore, retina of the human eye can be imaged with at least 100 times better axial resolution (Drexles & Fujimoto, 2008a) than that achievable using confocal microscopy. Such a high depth resolution is achievable even when imaging aberrated eyes and when using small diameter beams. This is one important aspect that contributed to the development of OCT, the decoupling of the achievable depth resolution from the value of the numerical aperture of the microscope objective. A high depth resolution image of the retina *in vivo* is shown in Figure 1 (Cucu *et al.,* 2006). A broadband source delivering a large bandwidth of 150 nm around 890 nm was employed, determining a depth resolution in the retina close to 3 μm. This exceptional high resolution allows identification of the layers labelled in Figure 1.

## Principles of operation

There are two main OCT methods, time domain (TD)-OCT and spectral domain (SD)-OCT (Drexler & Fujimoto, 2008). SD-OCT is attractive because it eliminates the need for depth scanning, which in TD-OCT is performed usually by mechanical means. SD methods can be implemented in two formats: (i) spectrometer based (SB) or (ii) by using a tunable laser or a swept source (SS). Each method, SB-OCT or SS-OCT has its own merits and deficiencies. The depth resolution achieved depends on the bandwidth of the optical source in the TD-OCT and SB-OCT and on the tuning bandwidth in the SS-OCT.

**Fig. 2:**
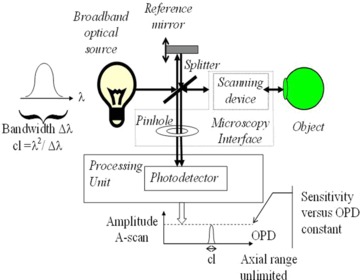
TD-OCT setup.

A TD-OCT setup is equipped with an optical source and an interferometer, as shown schematically in Figure 2 as a Michelson interferometer, where a *Reference Mirror* and an optical *Splitter* are used to produce a reference beam. A *Microscopy Interface* optics is employed to convey light from the *Splitter* to, and from the *Object* to be examined, up to a *Processing Unit*, that performs interference of light between the reference beam and the beam returned by the *Object*, as well as processing of the interference signal and its analysis. The path traversed by the object wave from the splitter to the object and back represents the object path length. The path traversed by the reference wave from the *Splitter* to the *Reference Mirror* and back represents the reference path length. An optical path difference (OPD) in the interferometer is defined as OPD =|object path length – reference path length|.

### TD-OCT

**Fig. 3:**
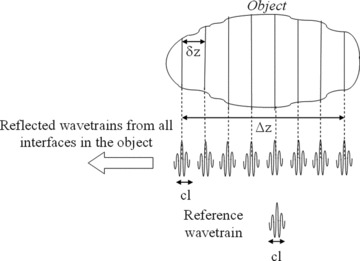
TD-OCT: Relative size of the coherence length, cl, required depth resolution δ*z*, and OPD axial range 2Δ*z* determined by the axial extension of the *Object* to be investigated. For simplicity, the index of refraction is considered as 1.

As shown in Figure 2, a TD-OCT setup uses a *Broadband optical source* and the *Processing Unit* employs a *Photodetector*. The principle of operation is based on partial coherence interferometry, where the *Photodetector* senses variations in the interference result as long as the OPD is less than the coherence length, cl, of the *Broadband source*. Let us say that the *Object* is made from a structure of layers at different depths, as illustrated in Figure 3. Each layer returns a replica of the incoming wavetrain, delayed correspondingly. By scanning the reference path length through moving the *Reference Mirror*, the layer satisfying the coherence gate condition, OPD < cl, can be selected. In Figure 3, the layer selected is that where its backscattered wavetrain is matched temporally by the reference wavetrain. Maxima of interference are obtained for each scatterer in depth that satisfies the condition OPD = 0. By scanning the OPD, a TD-OCT system outputs a reflectivity profile in depth, termed as an A-scan. So far, such a configuration is that of a low coherence interferometer widely used in sensing. OCT was invented by adding a transversal scanner to the object arm of a low coherence interferometer to scan the beam laterally over the object (Huang *et al.*, 1991). By collecting adjacent A-scans for successive pixels along a transversal coordinate, a cross-section image is obtained, termed as a B-scan (similar to the terminology used in ultrasound imaging). This imaging method has become what is now known as longitudinal (or axial) TD-OCT. Another version of TD-OCT is *en face* OCT, based on one-dimensional reflectivity profiles (T-scans), collected by flying the spot transversally, while maintaining the axial coordinate fixed (*Reference mirror* at rest). In this case, a B-scan image is constructed from many T-scans repeated for successive pixels in depth, in other words, scanning fast laterally and slow axially using the *Reference mirror*. An *en face* OCT system presents the advantage that it can collect a C-scan (constant depth) image in real time, by repeating the T-scans for adjacent values of the orthogonal lateral coordinate, for instance the T-scans are oriented along the *X*-coordinate and repeated along the *Y*-coordinate (Podoleanu & Rosen, 2008).

### SD-OCT

**Fig. 4:**
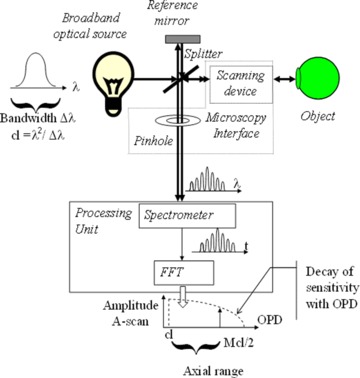
SB-OCT setup.

**Fig. 5:**
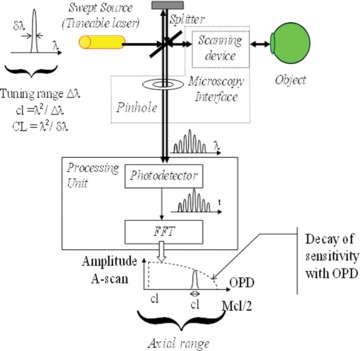
SS-OCT setup.

SD-OCT refers to spectral interrogation of the spectrum at the interferometer output. There are two possibilities, as illustrated in Figures 4 and 5. In Figure 4, a broadband optical source is used and the *Processing Unit* employs a spectrometer, usually built using a prism or a diffraction grating and a linear photodetector array, using a charged coupled device (CCD) or a complementary metal oxide semiconductor (CMOS) linear camera. In Figure 5, a *SS* (tunable laser) is used and the *Processing Unit* employs a *Photodetector*, similar to the TD-OCT setup in Figure 2. Mechanical scanning of the OPD in TD-OCT in Figure 2 is replaced by reading the charges on the array in the spectrometer in SB-OCT in Figure 4 or by tuning the frequency of the laser source in SS-OCT in Figure 5.

#### SB-OCT

**Fig. 6:**
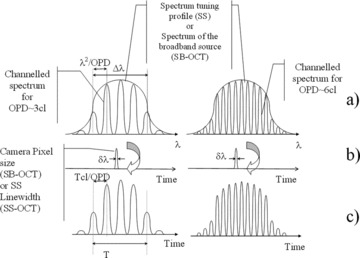
(a) Spectrum output of the interferometer for two OPD values, left: ∼3 cl, right: ∼6 cl; (b) SD-OCT sampling function, of width δλ, spectrum is read in a time *T* either by downloading the charge from the linear camera array, or by tuning the frequency of the SS, the large arrow suggests that by doing so, the spectrum shown above in (a) is transferred to the output signal shown in (c) below; (c) signal delivered by the *Processing unit* in Figures 4 and 5.

This method is an extension of the work on white light interferometry with initial applications in absolute ranging and sensing (Smith & Dobson, 1981). The operation of the SB-OCT is based on the optical spectrum output demodulation of the low coherence interferometer. The spectrum exhibits peaks and troughs (channelled spectrum) and the period of such a modulation is proportional to the OPD in the interferometer (Taplin *et al*., 1993). The larger the OPD, the larger the number of peaks in the spectrum, as shown in Figure 6(a) when using a mirror as *Object*, for an OPD of ∼3 cl (left) and ∼6 cl (right). The linear camera needs pixels of sufficient small size, δλ (Fig. 6b) to be able to sample the succession of peaks and troughs in the channelled spectrum. By downloading its charge content, the linear camera in the spectrometer transforms the optical spectrum into an electrical signal in time (Fig. 6c). If multilayered objects are imaged, such as retina or skin, each layer imprints its own spectrum modulation periodicity, depending on its depth. A fast Fourier transform of the linear camera signal translates the periodicity of the channelled spectrum (Fig. 6a) into peaks of different frequency, related to the OPD (Fig. 6c). Such a profile is essentially the A-scan profile of the square root of reflectivity in depth.

Due to its sensitivity advantage and availability of fast digital linear cameras, the SB-OCT became the method of choice in current OCT investigations of the retina with video-rate images from the retina demonstrated (Wojtkowski *et al.,* 2004). The majority of SB-OCT reports employ linear cameras at 20–70 kHz, which represents a line scan rate faster than maximum achievable by TD-OCT *en face* imaging using a resonant scanner at 16 kHz (Hitzenberger *et al.,* 2003) and more than 20 times faster than line scan rates in *en face* OCT using galvanometer scanners. Progress in multitap cameras allowed 312.5 kHz line rate for OCT images collected from the retina (Potsaid *et al.,* 2008).

#### SS-OCT

In Figure 5, the laser line, δλ, needs to be much narrower than the spectral distance between adjacent peaks, as shown in (Fig. 6 B). Figure 6(c) illustrates the signal output of the *Photodetector* in Figure 5, when tuning the SS. If in the ideal case, the laser line is approximated with a Dirac delta function (infinitesimally small linewidth δλ), then the photodetected signal takes the exact shape of the channelled spectrum. A fast Fourier transform of this signal translates the periodicity of the channelled spectrum into peaks of different frequency, related to the OPD. In this way, an A-scan is obtained, as shown at the bottom of Figure 5, when the *Object* is a mirror. Recent progress in the fast tunability of laser sources has revived the interest in SS-OCT. The time required to tune the wavelength determines the time to produce an A-scan. Tuning speeds in excess of 5 MHz (Wieser *et al*., 2010) makes the SS-OCT the fastest scanning OCT method that has lead to sufficient quality of *in vivo* images acquired from tissue to date (Klein *et al*., 2011).

## Comparison of TD-OCT and SD-OCT Methods

There is a fundamental difference between TD and SD methods. TD-OCT methods deliver the reflectivity of a single point in depth, at OPD = 0, whereas SD-low coherence interferometry (LCI) and SD-OCT return reflectivity values for all points along the axial range at once. This confers SD methods superiority in terms of acquisition rate. Despite this, the SD methods present some shortcomings, as detailed below. Table 1 summarises the parameters which determine the depth resolution and the axial range in each case, as well as other comparative features, with more details in (Podoleanu & Rosen 2008).

**Table 1:** Essential parameters in OCT

	*En face* TD-OCT	Longitudinal TD-OCT	SB-OCT	SS-OCT
Depth resolution is determined by:	Optical source bandwidth	Optical source bandwidth	Optical source bandwidth	Tuning bandwidth
Axial scan range is:	Unlimited	Unlimited	Limited by the spectrometer resolution	Limited by the optical source linewidth
Dynamic focus is:	Practical	Possible but difficult at high speed A-scan line rates	Nonapplicable	Nonapplicable
Sensitivity versus OPD is:	Constant	Constant	Maximum in OPD = 0	Maximum in OPD = 0
Mirror terms	None	None	Manifest if OPD = 0 is crossed	Manifest if OPD = 0 is crossed
Line rate determined by:	Transversal scanner	Axial scanner	Reading the linear camera	Tuning the swept source
Maximum achievable line rate	16 kHz (using a resonant scanner at 8 kHz)	∼100 kHz	∼300 kHz	∼5 MHz
Time to create an *en face* image of 500 lines each of 500 pixels along the line at the maximum achievable line rate is:	Given by the time to produce a C-scan frame, 31 ms	Given by the time to acquire a volume made from 500 B-scan frames of 500 line scans, ∼2.5 s	Given by the time to acquire a volume made from 500 B-scan frames of 500 line scans, ∼0.83 s	Given by the time to acquire a volume made from 500 B-scan frames of 500 line scans, ∼50 ms
Time to acquire a volume of 500 × 500 × 500 pixels at the maximum achievable line rate is:	15.5 s	∼2.5 s	∼0.83 s	∼50 ms

### Net advantage of SD-OCT in comparison with TD-OCT

#### Signal to noise ratio

Specialists from different disciplines investigated the performance of TD and SD interferometry approaches and provided insights into the fundaments of spectral processing from different angles. Mitsui (1999) has compared a TD-low coherence interferometer setup with a SB version. This has shown that the dynamic range of the SB implementation was larger than that of TD version by a factor given by the ‘number of saturation charges of the linear image sensor’ used in the spectrometer, i.e with the ‘number of spectral windows’.

### Disadvantages of SD-OCT in comparison with TD-OCT

#### Sensitivity versus depth

When employing TD principles, the same sensitivity is achievable for any OPD value, i.e. for any axial position of the scattering point along the A-scan, as shown at the bottom of Figure 2. In opposition, there is a decay of sensitivity of the SD methods with the OPD, as shown at the bottom of Figures 4 and 5. Both SB and SS methods require resolution in separating the channelled spectrum peaks and troughs, which translates into the need of large number of grating lines, *N*, and large number of pixel cameras, *M*, when using the SB-OCT method and of a narrow linewidth, δλ, for the SS used in the SS-OCT method. In fact, fundamental in limiting the axial range in both types of SD methods is the spatial extension of the interfering wavestrains, CL. This can be assimilated to an equivalent coherence length, hence the notation. In the SB, CL is given by the extension of the object and reference wavetrains after suffering dispersion (or diffraction) in the spectrometer. It should be noted that CL is much larger than the coherence length cl introduced above. In the SS-OCT case, the spatial extension, CL, of the wavetrains emitted by the swept source, is inverse proportional to the linewidth, δλ, of the instantaneas spectrum emitted.

**Fig. 7:**
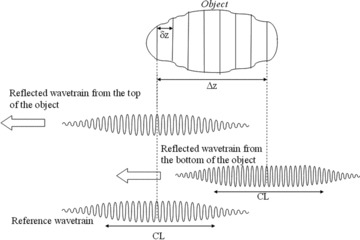
SD-OCT: Relative size of the CL extension of interfering wavetrains, required depth resolution δ*z*, and axial range Δ*z* determined by the axial extension of the *Object* to be investigated.

Figure 7 illustrates the spatial relativity of dimensions. The *Object* to be investigated is made of layers separated by δ*z* and the OPD axial extension of the object is 2Δ*z* (considering for simplicity the index of refraction as 1). In TD-OCT, the coherence length, cl, of the optical source needs to be smaller than δ*z* to be able to separate the layers in depth in the object, as shown in Fig. 3. Because TD-OCT operates around OPD = 0, by moving the *Reference Mirror* in the interferometer in Figure 2, maximum of sensitivity is moved from one layer in Figure 3 to the next. Therefore, all layers can be interrogated by the same sensitivity. This is not the case in SD-OCT.

To ensure interference of the local reference wave with both the waves coming from the top of the object as well as with the waves returned from the deepest layer, their spatial extension, CL, needs to exceed the OPD axial range 2Δ*z*, CL > 2Δ*z* as shown in Figure 7. Let us say that the *Reference mirror* is adjusted for OPD = 0 to match the top of the *Object*. As illustrated in Figure 7, total overlap of interfering wavetrains, object and reference, happens at OPD = 0 only, whereas for the maximum depth, tails only of object and reference wavetrains are superposed. The sensitivity depends on the amount of overlap of the two interfering wavetrains, object and reference, therefore, the sensitivity in SD-OCT decays with depth. However, because the wavetrains are longer than the axial range, 2Δ*z*, they will all interfere to some extent with the reference wavetrain. In this way, all OPD values within the axial range 2Δ*z* are interrogated at once. At any particular time, for any given optical frequency of the SS, wavetrains from all depths are returned back to the photodetector.

The wavetrain from the bottom of the object is shown with more cycles than the wavetrain from the top of the object, as the picture in Figure 7 represents a frozen aspect in time of returning wavetrains from the top and bottom of the object. (For clarity, the wavetrains from the top and bottom of the object are shown only and they are also displaced relative vertically, otherwise they should be in line, like in Figure. 3, and superposed.) Because of the frequency tuning, by the time the emitted wavetrain from the SS reaches the bottom of the object, the frequency of the wavetrains reaching the top of the object has changed. This difference in frequency will give the pulsation of the beating signal resulting from the interference of the object and reference wavetrains at the *Photodetector*. In this way, the axial position of scattering points along an A-scan in depth within the object is encoded on the frequency of the beating signal, with a linear proportionally between the frequency of the beating signal and the OPD value of scattering points.

In the case of the SB-OCT, the interfering waves are those after passing through a disperser, a prism or a diffraction grating. Their equivalent coherence length is elongated by dispersion/diffraction to CL. For instance, for a beam extending over *N* grating lines, the wavetrain length, CL in the first order of diffraction is *Nλ*. To cover an object of axial extension Δ*z*, for the reasons explained earlier, *Nλ* needs to be larger than Δ*z*. A second condition originates in the need that the camera in the spectrometer has sufficient number of *M* pixels to resolve the channelled spectrum. The number of cycles in the channelled spectrum is given approximately by the OPD/cl, where the coherence length, cl, can be approximated by 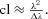 To resolve the largest number of peaks in the channelled spectrum, corresponding to the maximum axial range, where OPD = 2Δ*z*, and considering that two pixels are necessary to resolve a complete cycle of the *Photodetector* signal, the minimum number of pixels, *M*, on the camera needs to be: 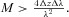 This is equivalent to requiring that the spectral width projected on each pixel, δλ, be shorter than half of the spectral distance 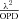 between adjacent peaks in the channelled spectrum for any given OPD (as illustrated in Fig. 6a). For the maximum axial range, OPD = 2Δ*z*, this condition becomes: 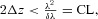 as commented above.

Similar conditions hold for SS-OCT, where δλ is the laser linewidth and *M* is the number of minimum equivalent steps within the tuning bandwidth, Δλ=*M*δλ/2. The division by 2 considers that at least two steps in the wavelength change per cycle in the channelled spectrum are required for proper sampling of the channelled spectrum.

In TD-OCT, for each position of the coherence gate, the two wavetrains overlap entirely, over all their cl extension (Fig. 3). In SD-OCT, the wavetrains overlap entirely over all their CL extension for OPD = 0 only, whereas for an increasing OPD value, their overlap reduces (Fig. 7). This explains why the sensitivity is constant with OPD in TD-OCT whilst it decays with OPD in SD-OCT as illustrated by the dashed curved at the bottom of Figures 4 and 5. To ensure a long CL and hence a large axial range 2Δ*z*, in SB-OCT, a large number of grating lines, *N* (and a large number of pixels *M* to sample the channelled spectrum in the array) are required, whereas in SS-OCT, a linewidth, δλ, as narrow as possible is needed.

Let us say that the spectrum of width Δλ spread over the linear camera in the spectrometer in the SB-OCT is read in a time, *T*. Equivalently, the frequency of the tuneable source in the SS-OCT is tuned within a spectrum of width Δλ in time, *T*. Then the frequency, *f*, of the signal at the output of *Processing unit* during either by reading the camera in SB-OCT in Figure 4 or by tuning the laser in SS-OCT in Figure 5 is: *f*= (1/*T*)(OPD/cl) where cl is the coherence length evaluated for the broadband spectrum Δλ in SB-OCT and evaluated for the tuning bandwidth Δλ in SS-OCT.

#### Mirror terms

In SD-OCT, the same cross-section image (upside down) results for the same modulus value of the OPD. If the OPD = 0 value is placed inside the tissue, then the image corresponding to a positive OPD is overlapped over the image for the negative OPD. In a SD-OCT system, the sensitivity profile is symmetric around OPD = 0, where it achieves a maximum. In order to avoid mirror terms corrupting the image, the OPD = 0 value is placed in front of the object examined, i.e. the object is displaced axially into a range of OPD values where sensitivity is less. Therefore an active line of research is focused on methods to attenuate the mirror terms. Several such methods rely on the generation of a complex interferogram, where its imaginary component is inferred by producing a second interferogram shifted in phase by π/2. Various techniques have been employed to generate such phase-shifted interferograms. Such methods allow correct reconstruction of layers in depth irrespective of the OPD sign and conserve the symmetric shape of sensitivity with OPD, with a maximum in OPD = 0 value. Such methods lead to doubling the axial OPD range and are therefore called full range methods (Bachmann *et al*., 2006). They advantageously require placing the OPD = 0 value inside the middle of the axial range of the object, conferring in this way, a higher sensitivity along the A-scan in comparison with conventional SD-OCT methods. A different method, applicable to SB-OCT, is based on Talbot bands (Bradu & Podoleanu, 2011). This, does not require phase shifts, no calculations or cancellation algorithms and therefore such a solution is not affected by sample movements or parameter instabilities. Simply put it, in Talbot bands configurations, mirror terms do not exist. In a Talbot bands configuration, the two interferometer beams are spatially separated in their way towards the disperser (prism or diffraction grating), separation that introduces a delay between the diffracted wave-trains equal or larger than their wave-train length, CL, after diffraction. This delay is conveniently used to shift the sensitivity curve versus OPD towards one sign of OPD values only.

#### Time to produce an en face C-scan slice and time to collect a volume

SB-OCT and SS-OCT setups output A-scans, therefore they cannot produce a 2D *en face* map (C-scan) image in real time. C-scan sections can be obtained in SB-OCT and SS-OCT only after a whole volume of the *Object* is acquired, i.e. via a postacquisition process only. In a first step, a series of B-scan OCT images is taken at different transverse coordinates to sample the whole volume, followed in a second step by sectioning the 3D volume so generated to slice a C-scan. Therefore, in SD-OCT, the time to produce a C-scan is determined by the time required to collect all volume data plus the time taken by the software cut. Recent progress in SS-OCT has lead to multi MHz A-scan line rates (Klein *et al.*, 2011). A 5 MHz line rate for instance allows a B-scan image of 500 lines to be created in 0.1 ms. If 500 such frames of 500 pixels in depth in the A-scan are acquired, this means a volume of 500^3^ of pixel data captured in approximately 0.05 s (Table 1). This represents the minimum time interval to acquire the data necessary to produce a C-scan image. The time interval necessary to practically produce a C-scan image is larger, as extra time is required for the software cut to sample the corresponding slice from the data volume. This shows the connection between the number of voxels acquired in the unit of time and the time taken before a C-scan image can be produced. Maximum acquisition speed in *en face* OCT is achievable using a resonant scanner at 16 kHz, in this case, a C-scan image of 500 lines can be created in 32 ms. Therefore, when compared with the 50 m-s above, *en face* TD-OCT can create a C-scan image slightly faster than the fastest SS-OCT systems reported so far. However, for a similar volume made of 500 pixels along 500 T-scans per C-scan in 500 C-scan frames, *en face* OCT requires 15.5 s, much longer than 50 ms taken by SS-OCT. Therefore, modern SS-OCT technology can create a volume with less movement artefacts than the TD-OCT technology. Multi MHz line rates coupled with parallel scanning can lead to even faster OCT rates in producing meaningful data volumes, as for instance in (Wieser *et al*., 2010) where a 5.4 MHz line rate with four simultaneous beams allowed collection of 4.5 GVoxels/s data.

#### Focus control and suitability of SD-OCT to image retina in the human eye

Another disadvantage of SD methods, especially when applied to OCT imaging with high transversal resolution, is that the focus control cannot be synchronized with the axial scanning. Such a capability, known as dynamic focus, can however be implemented in TD-OCT. This makes all methods of TD-OCT more attractive to microscopy applications and TD-*en face* OCT better suited to be combined with adaptive optics to visualize photoreceptors in the retina.

However, for imaging moving organs, such as the eye, SD-OCT methods are better suited. In this case, because all points along the A-scan need to be more or less in focus, a long depth of focus is needed. Fortunately, this condition is accomplished in the case of the retina in the human eye, where the retina in the macula region is 150 – 350 μm thick and the depth of focus of the human eye is over 300 μm. This is not the case with animal eyes, where the eye length is much smaller, in which case the thickness of the retina, Δ*z*, exceeds the depth of focus. In such cases, SD-OCT methods need to be repeated for different focus position to capture good images from all depths. A synthesized image can then be produced by using the parts of the cross-section images, which were in focus, according to a Gabor transformation based method proposed recently (Rolland *et al.*, 2010).

## Five important parameters of OCT

### Depth resolution

The larger the bandwidth of the source in TD-OCT and SB-OCT and the wider the tuning bandwidth in SS-OCT, the better the depth resolution is.

### Penetration depth

OCT relies on interference and therefore requires stable and deterministic phase relations between the interfering waves (Kramoreva & Rozhko, 2010). Due to scattering and absorption in the examined object, the number of photons in the backscattered wave conserving stable phase relations to the photons in the incident wave reduces with depth. The maximum penetration depth, Δ*z* is therefore determined by the depth wherefrom the object wave exhibits sufficient strength to achieve via interference, a signal equal to that of the noise.

### Amplification effect

OCT in fact operates like a homodyne or heterodyne radiofrequency receiver, where the weak signal from the object is beaten with the local reference signal, of much larger strength. The reference signal acts like a booster for the object wave. This effect can be assessed comparatively on human skin, where for instance at 800 nm, a confocal microscope, which relies on the wave from the object only, can achieve not more than 350–400 μm (Gambichler *et al*., 2011). For the same wavelength band, OCT can penetrate more than 1 mm in skin.

### Wavelength range

Biomedical optics requires imaging within the therapeutic window, situated between 600 and 1000 nm, where the main constituents of the tissue exhibit low absorption (Fercher *et al.*, 2003). The main window in imaging the retina is 800–900 nm, where water absorption is minimal. For imaging the anterior chamber, 800 nm as well as longer wavelengths, such as 1300 nm have been used. The 1020–1080 nm region of the near-infrared spectrum emerged as an attractive option for imaging the retina due to a relative minimum of the water absorption coefficient at 1060 nm (Hariri *et al.,* 2009). Development of nonmedical applications raised the interest of much longer wavelengths, suitable in special to objects with low water content.

### Power to the object

In scanning regime, safety standards allow: 0.7 mW at the cornea for imaging the retina of the human eye at 830 nm and up to 2 mW at 1060 nm (Hariri *et al*., 2009); 15 mW in imaging the anterior chamber at 1300 nm (Huang *et al.,* 2008) and larger power when imaging internal structures and skin (Tearney *et al*., 1997). Losses in splitters inside the OCT configuration require that the power delivered by the optical source be at least 2–10 times larger than the power to the tissue, limited by safety.

## Flying spot versus full-field imaging

Each OCT method can admit different versions of scanning and detection. Any OCT system is equipped with two or three scanning mechanisms. Flying spot implementations use galvo-scanners, resonant scanners, piezo-elements and acousto-optic modulators as *Scanning device* in Figures 2, 4 and 5 to deflect the beam over the *Object*, point by point. Full-field implementations use a linear photodetector array or a 2D array, a CCD or CMOS, to capture several points in the scene at once. In Figures. 2 and 5, when operating in full field, the scanning device between the splitter and the object is removed and the *Pinhole* and *Photodetector* are replaced with a 2D camera (Dubois *et al.*, 2004). In both cases, processing is performed on each camera pixel to return an A-scan while moving the *Reference Mirror* in Figure 2 or tuning the *SS* in Figure 4. In this way, the whole volume of the object is acquired. In Figure 4, full-field operation can be implemented by reducing the *Scanning device* to one direction deflector only, using cylindrical optics to illuminate the *Object* and replacing the linear photodetector array with a 2D CCD or CMOS array. For each position of the *Scanning device*, the 2D array delivers a cross-section image (a B-scan), in the plane of the line projected on the target and depth. Full-field versions are compact solutions, however with the disadvantage of crosstalk between pixels in the camera. Full field also provides an alternative for high speed acquisition in SS-OCT without the need of fast tuning rates for the SS. Access to high speed collection of 3D data can be either via increase in the sweep rate of the SS in the flying spot technology, or by combining a fast camera, such as a CMOS, with a slower SS, in full-field OCT where the tuning speed is dictated by the frame rate of the camera (Bonin *et al*., 2010). Such a combination has proved capable of producing sufficient quality images of the retina at 1.5 MHz line rate using a Y4 Redlake/IDT camera (IDT, Tallahassee, FL, USA).

## Applications

### Medical Applications

A Web of Knowledge search will show that more than half of the reports on OCT are in ophthalmology and optometry. OCT is now regularly used in diagnosis of a variety of diseases of the retina, in imaging and measurements of the anterior chamber and in the measurement of the eye length. In endoscopy, OCT prevails as the only technology capable of high resolution imaging, with almost 10 times better depth resolution than high frequency ultrasound (Patel *et al.,* 2005). Diagnosis and monitoring of a variety of skin conditions has been reported in dermatology (Gambichler *et al.,* 2011). Detailed reviews of work on laryngology, hard and soft dental tissues, pulmonary medicine, gynaecology, urology, examination of the human upper airways are found in (Drexler & Fujimoto, 2008b).

### Functional OCT

The OCT signal is intimately related to functional disturbances in the tissue, which usually precede morphological changes. Different versions of OCT systems provide functional information, such as polarisation sensitive OCT, spectrometric OCT and Doppler OCT.

Polarization-sensitive OCT (PSOCT) takes into account the vectorial nature of light waves. PSOCT can detect and quantify the polarization properties of the tissue by analyzing changes in the polarization state of the backscattered probe light beam. The information provided by PSOCT images can be used to identify birefringent structural constituents in the target tissue that are otherwise invisible to conventional OCT or other imaging techniques.

Since the first report of a time domain functional PSOCT system (Hee *et al*., 1992), a diversity of time domain and spectral domain configurations have been investigated. They all differ in complexity, capabilities and signal processing schemes. The most complete information about the polarization properties of a biological target is given by systems capable of producing depth resolved Mueller matrix elements (de Boer *et al*., 2002). These configurations can account for depolarization as well as changes in the linear and circular degree of polarization of the probe beam during propagation in tissue.

The retinal nerve fibre thickness is an essential parameter in the diagnosis of glaucoma and instruments based on retardation measurements infer this thickness by assuming that the double-pass phase retardation per unit of depth is the same throughout the eye fundus. PSOCT studies have shown that the retinal nerve fibre layer double-pass phase retardation per unit of depth (measured in degrees of rotation of the linear polarization vector/mm) varies across the retina between 0.18 and 0.37 degree/μm (Cense *et al*., 2004).

A change in the polarisation properties of tissue can be related to a change in the structure, functionality or integrity of the target. For instance, thermal injury denaturates collagen in skin and PSOCT can sense changes in the collagen birefringence (Todorovic *et al*., 2008). PSOCT can measure remineralization on dentin surfaces (Manesh *et al*., 2009) and abnormal birefringence in diseases of the cornea (Lim *et al*., 2011).

Spectroscopic OCT (SOCT) allows simultaneous OCT measurements in multiple band windows. SOCT can provide information on the oxygenation or on the concentration of specific constituents of the tissue by exploiting their spectral absorption behavior. The larger the number of interrogating wavelengths the better the quantitative analysis. The availability now of large bandwidth sources (Robles *et al*., 2012) allows implementation of SOCT to provide depth resolved distribution of haemoglobin in tissue, not technologically possible using combinations of discrete sources.

Doppler OCT (DOCT) can be used to measure or monitor Brownian motion and flows of biologic liquids. This method provides a depth resolved profile of the flow velocity in the vessel, with the resolution determined by the coherence length of the source. DOCT microangiography can provide enhanced visualization of retinal and choroidal vasculature (Jaillon *et al*., 2011). DOCT has potential as a noninvasive quantitative method to measure tissue perfusion over a physiologic range, in studies of cerebrovascular physiology (Srinivasan *et al*., 2011). DOCT can also be used in the assessment of tissue deformation, to determine the biomechanical characteristics of the embryonic heart (Li *et al*., 2011).

### Nonmedical applications

A review of nonbiomedical OCT (Stifter, 2008) presents the OCT capabilities in areas such as dimensional metrology referring to surface topography, thickness measurements, simultaneous measurement of thickness and indices of refraction, measurement of defects in ceramics and glass, imaging and characterisation of photoresist mould miniature gear wheels, of porous structure of polymer foam samples, fibre composites and paper. In art conservation, OCT has been applied for assessment of the quality and thickness of the varnish layer over paint layers on paintings, ceramics, identification of underdrawings and topography measurements of surfaces (Hughes, 2010). In dentistry, it was found useful in the characterisation of gaps between different materials employed in dental constructs, such as polymers, ceramics and metal (Sinescu *et al.*, 2008).

## Conclusions

Twenty years after the introduction of the OCT (Huang *et al*., 1991), advancement of OCT technology continues to be an active field of research. In great proportion, the progress in recent years is due to the continuous attention given to the field of optical sources for OCT. This is somehow expected due to the essential dependence of OCT performance to the optical source in terms of depth resolution. In SS-OCT, the source determines not only the depth resolution, but also the scanning protocols and the acquisition speed. Fast SSs, based on a variety of principles such as that of Micro-Electro-Mechanical Systems (MEMS)/Vertical cavity surface emitting lasers (VCSELs) (Jayaraman *et al.*, 2011), ring lasers equipped with a fast tuning filter (Wieser *et al*., 2010) and dispersive cavity mode-locked lasers (Lee *et al.*, 2011) have been reported with tuning rates exceeding hundred of kHz making the SS-OCT method the fastest.

Novel concepts have been put forward, which straddle the delimitation between the optical source and the optical configuration. For instance, by stretching the supercontinuum and using a dispersive element, such as a dispersion compensating fibre, a SS is obtained. By using Raman amplification with dispersion compensating fibre in both interferometer arms (Goda *et al*., 2008), the fastest A-scan rate was reported, of 36 MHz.

Another focus is the combination of OCT with other imaging techniques and combination of OCT with assistive technologies such as adaptive optics and tracking (Podoleanu & Rosen, 2008). Any flying spot OCT is assembled around a confocal microscope and therefore, several versions of combined technologies have been reported for dual imaging systems, OCT/confocal microscopy, some providing three channels, with a 3rd channel tuned on fluorescence. A typical example is that of combining OCT with scanning laser ophthalmoscopy (SLO), that resulted in the imaging instrument known as OCT/SLO for imaging the eye. This was extended from TD-*en face* OCT, where both images where C-scans to SD-OCT, where the SLO channel continues to provide a C-scan whereas the OCT channel delivers a B-scan along selected lines from the SLO image.

Combination of assistive techniques with OCT improves the operation of the OCT channel and enhances the accuracy of the information provided. Techniques of adaptive optics corrected wavefronts have been incorporated into both TD and SD-OCT systems with applications in ophthalmology and microscopy. Tracking proved essential in imaging the eye to take advantage of the high resolution achievable with OCT (Podoleanu & Rosen 2008).

A present trend is in combining OCT with photoacoustics, as a complementary technique (Zhang *et al.*, 2010). OCT delivers a structure image, based on discontinuities in the real part of the indices of refraction at interfaces. Photoacoustics is sensitive to absorption changes, therefore providing images sensitive to the imaginary part of the index of refraction, and in this way, adding information on function to that of structure provided by the OCT.

For its advantage of speed, it is envisaged that SD-OCT will dominate the imaging of moving organs and is more suitable for hand held probes. Where transversal resolution is paramount and the object is stationary, then TD-OCT is superior, due to its compatibility with dynamic focus. There are applications, such as in microscopy, where the C-scan mode is favoured, in which case, *en face* OCT is the most suitable, meeting simultaneously, criteria of image orientation, high transversal resolution, dynamic focus and compatibility with adaptive optics. Where the *en face* view is essential, full-field TD-OCT is also recommendable, as for applications in histology. More progress is expected in the full-field SS-OCT due to the achievable more compact volume of its implementations and due to the evolution in the fields of SSs and fast cameras. Therefore, both approaches, flying spot and full field will coexist and continue to evolve. Due to the fact that no method meets all criteria related to resolution and acquisition speed, it is also expected that both TD and SD directions will continue to evolve.

## References

[b30] Bachmann AH, Leitgeb RA, Lasser T (2006). Heterodyne Fourier domain optical coherence tomography for full range probing with high axial resolution. Opt. Express.

[b1] Bonin T, Franke G, Hagen-Eggert M, Koch P, Hüttmann G (2010). In vivo fourier-domain full-field oct of the human retina with 1.5 million a-lines/s. Opt. Lett.

[b2] Bradu A, Podoleanu AGh (2011). Attenuation of mirror image and enhancement of the signal-to-noise ratio in a Talbot bands optical coherence tomography system. J. Biomed. Opt.

[b31] Cense B, Chen TC, Park BH, Pierce MC, de Boer JF (2004). In vivo birefringence and thickness measurements of the human retinal nerve fibre layer using polarization-sensitive optical coherence tomography. J Biomed Opt.

[b3] Cucu RG, Podoleanu AGh, Rogers JA, Pedro J, Rosen RB (2006). Combined confocal scanning ophthalmoscopy/en face T-scan based ultrahigh resolution OCT of the human retina in vivo. Opt. Lett.

[b32] Drexler W, Fujimoto JG (2008a). State-of-the-art retinal optical coherence tomography. Progress in retinal and eye research.

[b4] Drexler W, Fujimoto J (2008b). Series: Biological and Medical Physics, Biomedical Engineering, XXVIII. *Optical Coherence Tomography Technology and Applications*.

[b5] Dubois A, Grieve K, Moneron G, Lecaque R, Vabre L, Boccara C (2004). Ultrahigh-resolution full-field optical coherence tomography. Appl. Opt.

[b6] Fercher AF, Drexler W, Hitzenberger CK, Lasser T (2003). Optical coherence tomography – principles and applications. Rep. Prog. Phys.

[b7] Gambichler T, Jaedicke V, Terras S (2011). Optical coherence tomography in dermatology: technical and clinical aspects. Arch. Dermatol. Res.

[b8] Goda K, Solli DR, Jalali B (2008). Real-time optical reflectometry enabled by amplified dispersive Fourier transformation. Appl. Phys. Lett.

[b9] Hariri S, Moayed AA, Dracopoulos A, Hyun C, Boyd S, Bizheva K (2009). Limiting factors to the OCT axial resolution for *in-vivo* imaging of human and rodent retina in the 1060 nm wavelength range. Opt. Express.

[b33] Hee MR, Huang D, Swanson EA, Fujimoto JG (1992). Polarization-sensitive low-coherence reflectometer for birefringence characterization and ranging. JOSAB.

[b10] Hitzenberger CK, Trost P, Lo P, Zhou Q (2003). Three-dimensional imaging of the human retina by high-speed optical coherence tomography. Opt. Express.

[b11] Huang D, Swanson EA, Lin CP (1991). Optical coherence tomography. Science.

[b12] Huang D, Li Y, Tang M, Drexler W, Fujimoto J (2008). Series: Biological and Medical Physics, Biomedical Engineering, XXVIII. *Optical Coherence Tomography Technology and Applications*.

[b13] Hughes M (2010). Optical coherence tomography for art conservation and archaeology: methods and applications.

[b34] Jaillon F, Makita S, Min EJ, Lee BH, Yasuno Y (2011). Enhanced imaging of choroidal vasculature by high-penetration and dual-velocity optical coherence angiography. Biomed. Opt. Express.

[b14] Klein T, Wieser W, Eigenwillig CM, Biedermann BR, Huber R (2011). Megahertz OCT for ultrawide-field retinal imaging with a 1050 nm Fourier domain mode-locked laser. Opt. Express.

[b15] Kramoreva LI, Rozhko YuI (2010). Optical coherence tomography (Review). J. Appl. Spectrosc.

[b16] Lee HW, Lee JH, Jeong MY, Kim C-S (2011). Characterization of wavelength-swept active mode locking fiber laser based on reflective semiconductor optical amplifier. Opt. Express.

[b35] Li P, Liu AP, Shi L, Yin X, Rugonyi S, Wang RK (2011). Assessment of strain and strain rate in embryonic chick heart in vivo using tissue Doppler optical coherence tomography. Phys Medicine Biol.

[b37] Lim Y, Yamanari M, Fukuda S, Kaji Y, Kiuchi T, Miura M, Oshika T, Yasuno Y (2011). Birefringence measurement of cornea and anterior segment by office-based polarization-sensitive optical coherence tomography. Biomed. Opt. Express.

[b40] Manesh SK, Darling CL, Fried D (2009). Polarization-sensitive optical coherence tomography for the nondestructive assessment of the remineralization of dentin. J. Biomed. Opt.

[b17] Mitsui T (1999). Dynamic range of optical reflectometry with spectral interferometer. Jpn. J. Appl. Phys.

[b18] Patel NA, Stamper DL, Brezinski ME (2005). Review of the ability of optical coherence tomography to characterize plaque, including a comparison with intravascular ultrasound. Cardiovasc. Intervent. Radiol.

[b19] Podoleanu AGh, Rosen RB (2008). Combinations of techniques in imaging the retina with high resolution. Prog. Retin. Eye Res.

[b20] Potsaid B, Gorczynska I, Srinivasan VJ, Chen Y, Jiang J, Cable A, Fujimoto JG (2008). Ultrahigh speed Spectral/Fourier domain OCT ophthalmic imaging at 70,000 to 312,500 axial scans per second. Opt. Express.

[b69] Robles FE, Wilson C, Grant G, Wax A (2011). Molecular imaging true-colour spectroscopic optical coherence tomography. Nature Photonics.

[b21] Rolland JP, Meemon P, Murali S, Thompson KP, Lee K-S (2010). Gabor-based fusion technique for Optical Coherence Microscopy. Opt. Express.

[b22] Sinescu C, Negrutiu ML, Todea C (2008). Quality assessment of dental treatments using en-face optical coherence tomography. J. Biomed. Opt.

[b23] Smith LM, Dobson CC (1981). Absolute displacement measurements using modulation of the spectrum of white light in a Michelson interferometer. Appl. Opt.

[b38] Srinivasan VJ, Atochin DN, Radhakrishnan H, Jiang JY, Ruvinskaya S, Wu C, Barry S, Cable AE, Ayata C, Huang PL, Boas DA (2011). Optical coherence tomography for the quantitative study of cerebrovascular physiology. J cerebral Blood Flow Metabolism.

[b24] Stifter D (2008). Beyond biomedicine: a review of alternative applications and developments for optical coherence tomography. Appl. Phys. B: Lasers Opt.

[b25] Taplin S, Podoleanu AGh, Webb DJ, Jackson DA (1993). Displacement sensor using channeled spectrum dispersed on a linear CCD array. Electron. Lett.

[b26] Tearney GJ, Brezinski ME, Bouma BE, Boppart SA, Pitris C, Southern JF, Fujimoto JG (1997). In Vivo endoscopic optical biopsy with optical coherence tomography. Science.

[b39] Todorovic M, Jiao S, Ai J, Pereda Cubián,D, Stoica G, Wang LV (2008). “In vivo burn imaging using Mueller optical coherence tomography”. Opt. Express.

[b27] Wieser W, Biedermann BR, Klein T, Eigenwillig CM, Huber R (2010). Multi-megahertz OCT: high quality 3D imaging at 20 million a-scans and 4.5 GVoxels per second. Opt. Express.

[b28] Wojtkowski M, Srinivasan VJ, Ko TH, Fujimoto JG, Kowalczyk A, Duker JS (2004). Ultrahigh-resolution, high-speed, Fourier domain optical coherence tomography and methods for dispersion compensation. Opt. Express.

[b29] Zhang EZ, Laufer J, Povazay B, Alex A, Hofer B, Drexler W, Beard P (2010). Multimodal simultaneous photoacoustic tomography, optical resolution microscopy and OCT system. Proc. SPIE.

